# Curcumin Modulates Pancreatic Adenocarcinoma Cell-Derived Exosomal Function

**DOI:** 10.1371/journal.pone.0132845

**Published:** 2015-07-15

**Authors:** Carlos J. Diaz Osterman, James C. Lynch, Patrick Leaf, Amber Gonda, Heather R. Ferguson Bennit, Duncan Griffiths, Nathan R. Wall

**Affiliations:** 1 Division of Biochemistry, Center for Health Disparities and Molecular Medicine, Loma Linda University, Loma Linda, California, United States of America; 2 Department of Anatomy, Loma Linda University School of Medicine, Loma Linda, California, United States of America; 3 Malvern Instruments Inc., Rancho Cucamonga, California, United States of America; University of South Alabama, UNITED STATES

## Abstract

Pancreatic cancer has the highest mortality rates of all cancer types. One potential explanation for the aggressiveness of this disease is that cancer cells have been found to communicate with one another using membrane-bound vesicles known as exosomes. These exosomes carry pro-survival molecules and increase the proliferation, survival, and metastatic potential of recipient cells, suggesting that tumor-derived exosomes are powerful drivers of tumor progression. Thus, to successfully address and eradicate pancreatic cancer, it is imperative to develop therapeutic strategies that neutralize cancer cells and exosomes simultaneously. Curcumin, a turmeric root derivative, has been shown to have potent anti-cancer and anti-inflammatory effects *in vitro* and *in vivo*. Recent studies have suggested that exosomal curcumin exerts anti-inflammatory properties on recipient cells. However, curcumin’s effects on exosomal pro-tumor function have yet to be determined. We hypothesize that curcumin will alter the pro-survival role of exosomes from pancreatic cancer cells toward a pro-death role, resulting in reduced cell viability of recipient pancreatic cancer cells. The main objective of this study was to determine the functional alterations of exosomes released by pancreatic cancer cells exposed to curcumin compared to exosomes from untreated pancreatic cancer cells. We demonstrate, using an *in vitro* cell culture model involving pancreatic adenocarcinoma cell lines PANC-1 and MIA PaCa-2, that curcumin is incorporated into exosomes isolated from curcumin-treated pancreatic cancer cells as observed by spectral studies and fluorescence microscopy. Furthermore, curcumin is delivered to recipient pancreatic cancer cells via exosomes, promoting cytotoxicity as demonstrated by Hoffman modulation contrast microscopy as well as AlamarBlue and Trypan blue exclusion assays. Collectively, these data suggest that the efficacy of curcumin may be enhanced in pancreatic cancer cells through exosomal facilitation.

## Introduction

Currently, pancreatic cancer is one of the most devastating diagnoses to receive. It is responsible for one of the highest mortality rates among cancer types [1]. It is anticipated that 48,960 people will be diagnosed with pancreatic cancer and 40,560 people will die from pancreatic cancer in the United States this year [2]. These unacceptably high mortality rates are linked to inadequate screening tools and therapeutic options, as well as the aggressive nature of the disease [3, 4]. This aggressiveness is correlated with the influence of the tumor microenvironment, which is composed of blood vessels, immune cells, fibroblasts, extracellular matrix and cancer cells [5]. The signaling networks between the components of the tumor microenvironment are important drivers of tumor growth. Investigations have shown that tumor-derived extracellular vesicles such as exosomes are key modulators of this communication due to their capacity to transport cancer-promoting material [6–9]. In order to successfully treat pancreatic cancer, it is crucial to develop novel therapeutic strategies that concomitantly target tumor cells and important mediators of the tumor microenvironment such as exosomes.

Curcumin is a turmeric root derivative that has been considered as a potential pancreatic cancer therapeutic agent. Studies have demonstrated lower cancer incidence in countries with high curcumin consumption [10, 11] and preclinical studies have shown that curcumin exhibits anti-cancer [12–18] and anti-inflammatory properties in different cancer types *in vitro* and *in vivo*. Additionally, curcumin has synergistic effects with Gemcitabine, the gold standard treatment for pancreatic cancer [19–21]. These encouraging results have prompted researchers to assess the efficacy of curcumin in the treatment of pancreatic cancer. Phase I and II clinical trials have yielded promising results on the use of curcumin as part of pancreatic cancer therapeutic strategies [22–27]. However, curcumin has low bioavailability and this is one of the major obstacles to its application in the clinical setting. To overcome this, investigations have moved toward the development of innovative delivery approaches for curcumin, such as liposomes and nanoparticles, to enhance its bioavailability and efficacy [26, 28–36]. It has been demonstrated in various cancer types, including pancreatic cancer, that curcumin’s solubility and efficacy is enhanced by liposomal delivery *in vitro* and *in vivo* with minimal toxicity, providing promising evidence for clinical application [35–38]. Studies performed with nanoparticle-based curcumin, Theracurmin, have indicated that membrane encapsulation can improve the bioavailability of hydrophobic compounds such as curcumin by increasing water solubility [32, 34]. Furthermore, phase I studies with Theracurmin demonstrated that this treatment approach is non-toxic and results in higher curcumin bio-distribution compared to non-encapsulated curcumin in patients with pancreatic cancer [26, 34, 39]. In addition to being encapsulated in synthetic nanoparticles, curcumin is able to be packaged in lymphoma-derived exosomes and retain its anti-inflammatory function after delivery to recipient cells [40].

Collectively, these data suggest that curcumin is a suitable candidate for pancreatic cancer therapy due to its anti-cancer and anti-inflammatory properties. Moreover, curcumin has the potential to influence the role of exosomes in the tumor microenvironment. Thus, the objective of this study was to evaluate the impact of exosomal curcumin on recipient pancreatic cancer cells. The present study demonstrates, for the first time, that exosomes from curcumin-treated pancreatic cancer cells carry curcumin and that these curcumin-containing exosomes reduce the viability of recipient pancreatic cancer cells. These findings suggest that the effects of curcumin may extend to other components of the tumor microenvironment through exosomes.

## Materials and Methods

### Cells and culture conditions

The pancreatic adenocarcinoma cell line PANC-1 was acquired from the American Type Culture Collection (ATCC, catalog no. CRL-1469, Manassas, VA) and maintained in Dulbecco’s modified Eagle medium (DMEM; ATCC, Manassas, VA) supplemented with Normocin at a final concentration of 100 μg/mL (InvivoGen, San Diego, CA), 100 units of penicillin, 100 μg/mL of streptomycin, 300 μ/mL of L-glutamine and 10% USDA-sourced heat-inactivated fetal bovine serum (Mediatech, Manassas, VA). The pancreatic adenocarcinoma cell line MIA PaCa-2 was also acquired from ATCC (catalog no. CRL-1420) and maintained in DMEM (Mediatech) supplemented with 2.5% horse serum, 100 μg/mL Normocin (Invivogen), 100 units of penicillin, 100 μg/mL of streptomycin, 300 μ/mL of L-glutamine and 10% USDA-sourced heat-inactivated fetal bovine serum (Mediatech). In all the experiments, cells were cultured at 37°C in a humidified atmosphere containing 5% CO_2_ to 70–80% confluency prior to use.

### Preparation of solutions

Curcumin (Sigma Aldrich, St. Louis, MO) stock solutions (13.5mM) were prepared using DMSO and ethanol as solvents. Subsequent dilutions were made from this stock solution in fully supplemented DMEM to a final concentration of 50 μM. Heparin sodium salt (Sigma Aldrich) was used to prepare a 50 mg/mL stock solution in sterile water and used at a final concentration of 10 μg/mL. Recipient PANC-1 cells were pre-treated with 10 μg/mL heparin in fully supplemented DMEM for 30 minutes at 37°C, 5% CO_2_ prior to incubation with exosomes. Of note, 10 μg/mL heparin was also added during the subsequent incubation with exosomes.

### Exosome isolation

Exosomes were isolated from conditioned media as previously described by Savina et al. [41] with minor modifications. Briefly, PANC-1 or MIA PaCa-2 cells were cultured in fifteen T75 flasks at 1.5–2.0 x 10^6^ cells per flask and conditioned media (CM) was collected following 24 hours of treatment with fully supplemented DMEM (for isolation of curcumin-negative exosomes) or 50 μM of curcumin (for curcumin-positive exosome isolation). The cellular debris and other impurities in the CM were eliminated by three consecutive cycles of centrifugation. First, the CM was centrifuged in a Beckman Coulter Allegra X-15R centrifuge (SX475OA rotor) at 400 x g for 10 minutes, then at 2000 x g for 20 minutes, followed by centrifugation in a Thermo Scientific Sorvall Legend X1R centrifuge (F15-8X50Y rotor) at 10,000 x g for 30 minutes. Subsequently exosomes were isolated from the CM by ultracentrifugation in a Beckman XL-90 centrifuge equipped with a SW-27 rotor at 24,000rpm for 16 hours at 4°C over a 30% sucrose cushion. The exosomes within the sucrose cushion were washed with 1X PBS and centrifuged in a Beckman XL-90 centrifuge equipped with a 70-Ti rotor at 31,000 rpm for 2 hours at 4°C. The remaining pellet, the exosomal fraction, was resuspended in 500 μL of 1X PBS, transferred to a microcentrifuge tube, and immediately used in subsequent assays.

### Exosome detection and validation

To confirm exosome isolation, known methods of exosome detection, sizing and quantification were employed. Since it has previously been established that the acetylcholinesterase enzyme is enriched in exosomes [42], an acetylcholinesterase activity assay was used to detect the presence of exosomes in our isolates based on the protocols described by Savina et al. and Lancaster and Febbrario [41, 43] with minor modifications. Briefly, 37.5 μL of the exosomal fraction were transferred into each of three wells of a 96-well flat-bottom plate. 112.5 μL of 1.25 mM acetylthiocholine (Sigma) and 150 μL of 0.1 mM 5,5’-dithio-bis(2-nitrobenzoic acid) (Sigma) were then added to each well. The samples were immediately analyzed using a μQuant spectrophotometer (Bio-Tek Inc., Winooski, VT) and changes in absorbance at 412 nm were monitored every 5 minutes for 30 minutes. The exosome absorbance data were analyzed using the KC Junior software (Bio-Tek Inc.). The results presented represent acetylcholinesterase enzymatic activity after 30 minutes compared to control (reagents in assay diluent, 1X PBS).

Nicomp 380 ZLS analysis (Particle Sizing Systems, Port Richey, FL) was used to assess the size of the particles present in the exosome isolation fraction. Briefly, the exosomal fraction was diluted 1:30 with 1X PBS in a 4 mL (1 cm x 1 cm) plastic cuvette at 23°C and the size dispersion was measured using Nicomp 380 ZLS dynamic light scattering (DLS) with a display range of 0.6 to 6000 nm. The sample was exposed to a HeNe 5 mW laser using a wavelength of 639 nm. The data were analyzed using the Nicomp Fit Model Type and PSS zpw 388 Nicomp software. Finally, real-time detection, sizing, and quantification were performed using a NanoSight NS300 following the manufacturer’s protocols (Malvern Instruments, Malvern, UK). Briefly, exosome isolates were diluted 1:1000 in 1X PBS and sonicated in a water bath sonicator for 30 seconds to prevent exosome aggregation. Samples were then loaded into the NS300 instrument and subjected to nanoparticle tracking analysis (NTA) yielding size distribution and concentration (particles/mL). Acetylcholinesterase activity assays and NanoSight NTA were performed in three independent experiments, while Nicomp DLS analysis was performed in two independent experiments.

### Spectral studies

The spectral properties of curcumin, particularly a characteristic absorbance peak at 420 nm, have been utilized in detecting the compound under experimental conditions. These spectral analyses of exosomal curcumin were designed, with minor modifications, based on studies detecting curcumin within cells [44–46]. Briefly, exosomes were isolated as described above. To determine whether curcumin was coating the exterior surface of exosomes, exosomal fractions were subjected to spectral analysis prior to methanol-sonication disruption of exosomal membranes. To determine whether curcumin was located in the interior of the exosomes, the exosomal fraction was resuspended in 1 mL of 100% methanol and sonicated to disrupt exosome membrane integrity. The samples were then centrifuged at 10,000 rpm for 5 minutes at 4°C and the supernatants were collected for absorbance analysis at 420 nm using a μQuant spectrophotometer equipped with KC Junior software. Exosomes from untreated cells (curcumin-negative exosomes) were also isolated and lysed in methanol and subjected to spectral analysis to determine baseline auto-fluorescence of exosomes without curcumin. 1X PBS (for non-lysed exosome samples) or methanol (for lysed exosome samples) were used as negative controls for this assay. Data are representative of three independent experiments.

### Fluorescence imaging

Fluorescence microscopy was performed to detect the entry of exosomal curcumin into recipient PANC-1 cells. Briefly, PANC-1 cells were cultured in 6-well plates containing sterile cover slips at 3.0 x 10^5^ cells per well and exposed to curcumin-negative or curcumin-positive exosomes for 24 hours. Subsequently, cells were washed three times with 1X PBS, followed by fixation with 4% paraformaldehyde overnight at -20°C and permeabilization using 0.1% Igepal in 1X PBS for 10 minutes at room temperature. The cover slips were then washed three times with 1X PBS and placed onto slides with the nuclear stain DAPI in mounting medium for 5 minutes. Stained slides were imaged using a BZ-9000 BIOREVO fluorescence microscope (Keyence, Itasca, IL) with a 40X magnification objective. In a separate experiment, PANC-1 cells were incubated with 10 μg/mL of heparin for 30 minutes prior to exposure to curcumin-positive exosomes to determine exosomal curcumin delivery in the presence of heparin, an inhibitor of exosomal uptake by recipient cells [47–49]. Results are representative of three independent experiments; within each experiment, three images were acquired from different portions of each slide to obtain a representative image. Quantification of curcumin fluorescence per cell was performed using the BZ II analyzer software (Keyence, Itasca, IL). For each image acquired (three per independent experiment, three independent experiments), the hybrid cell count and adjust extension area software features were used to calculate cell number based on DAPI staining, followed by quantification of curcumin fluorescence. For each image acquired, curcumin fluorescence was divided by cell number and multiplied by 100 and the averages of these values were obtained for three independent experiments. Graphs were generated using the Prism (Graphpad, La Jolla, CA) software.

### Cell viability

Cell viability following exosomal curcumin entry was detected using AlamarBlue and Trypan blue exclusion assays. Briefly, PANC-1 or MIA PaCa-2 cells were cultured in 96-well flat-bottom plates at 1.0 x 10^4^ cells per well in the presence of fully supplemented DMEM (untreated), curcumin-negative exosomes, or curcumin-positive exosomes for 24, 48 and 72 hours. Cellular morphology was monitored at each time point using Hoffman modulation contrast microscopy and imaged using an Olympus IX70 microscope equipped with an Insight Spot 2 Mega Sample camera and software. Three independent experiments were performed and within each experiment, three images were captured in different sections to obtain a representative image. Cell viability was evaluated using AlamarBlue and Trypan blue exclusion assays. For AlamarBlue assays, the AlamarBlue reagent (Life Technologies, Grand Island, NY) was added to each sample at a 10% final concentration and incubated at 37°C/5% CO_2_ for 2 hours. Viability was analyzed by detection of absorbance at 570 nm using 600 nm as a reference wavelength in a μQuant spectrophotometer. For Trypan blue exclusion assays, cells were trypsinized and combined with the Trypan blue reagent (Life Technologies) and subjected to automated assessment using a TC-20 automated cell counter (Bio-Rad Laboratories, Inc., Hercules, CA). To determine whether any observed changes in viability were due to exosome-mediated effects, a separate sample was pre-treated with 10 μg/mL heparin for 30 minutes prior to addition of curcumin-positive exosomes; heparin was maintained during subsequent treatment with curcumin-positive exosomes. Results presented are representative of three independent experiments and each treatment (curcumin-negative exosomes, curcumin-positive exosomes, and heparin + curcumin-positive exosomes) is normalized to the untreated control.

### Statistical analysis

All statistical analyses in this study were performed using either one-way ANOVA or Students’ t-test analyses using the Prism (Graphpad) software. Statistical analysis of acetylcholinesterase activity assays was performed using Kruskal-Wallis one-way ANOVA with a post-hoc Dunn’s multiple comparison test. Analysis of curcumin fluorescence was performed using an unpaired one-tailed Students’ t-test. Statistical analysis of viability assays was performed using one-way ANOVA with a post-hoc uncorrected Fisher’s LSD test. A probability of less than a 95% confidence limit (p < 0.05) was considered to be significant. Data are presented as mean + standard error of the mean (SEM).

## Results

### Detection, size distribution and quantification of exosomal particles

**Fig 1:**
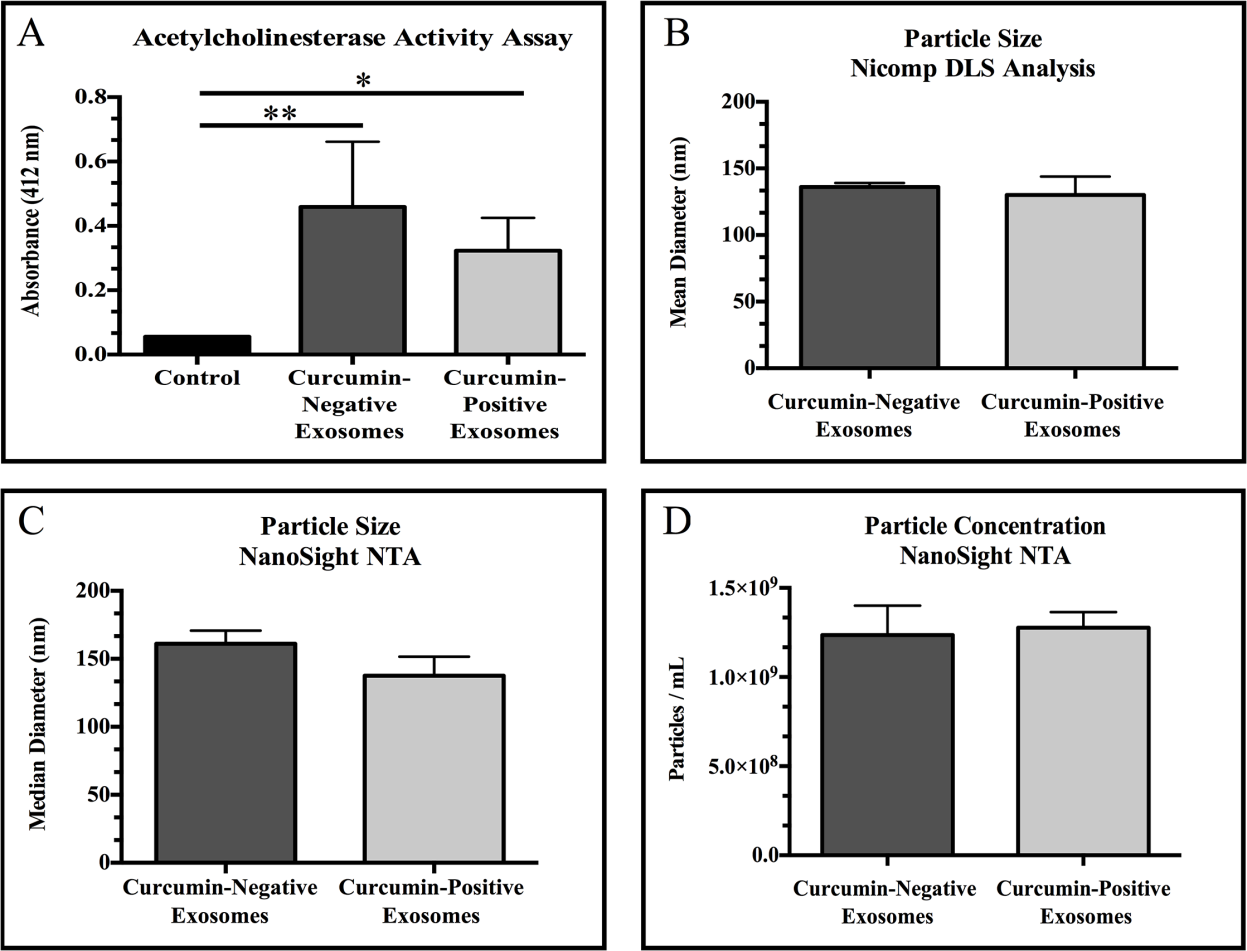
Validation of exosome isolation. (A) Acetylcholinesterase activity assays were used to detect exosomes in isolates from untreated PANC-1 cells (curcumin-negative exosomes) or PANC-1 cells treated with 50 μM of curcumin for 24 hours (curcumin-positive exosomes) compared to assay diluent, 1X PBS (control). (B) Nicomp dynamic light scattering (DLS) analysis was used to measure size distribution of particles in exosome isolates. (C) NanoSight nanoparticle tracking analysis (NTA) was used to confirm size distribution of particles in exosome isolates. (D) Particle concentration (particles/mL) was measured using NanoSight NTA. No significant differences were observed in acetylcholinesterase activity, size distribution, or particle concentration between curcumin-negative exosomes and curcumin-positive exosomes. Data are represented as mean ± SEM of three independent experiments, *p<0.05, **p<0.01, exosome fraction versus control.

Our data demonstrate significantly increased acetylcholinesterase activity in exosome isolates compared to control (assay reagents in 1X PBS; p = 0.0107, curcumin-negative exosomes versus control; p = 0.0171, curcumin-positive exosomes versus control) (Fig 1A). No significant difference was observed in acetylcholinesterase activity between exosomes derived from untreated PANC-1 cells (curcumin-negative exosomes) and exosomes derived from PANC-1 cells treated with 50 μM of curcumin (curcumin-positive exosomes) (p = 0.9999). To further validate exosome isolation, particle size was measured using two separate methods: Nicomp dynamic light scattering (DLS) analysis and NanoSight nanoparticle tracking analysis (NTA). Our data demonstrate that both exosome isolates (curcumin-negative exosomes and curcumin-positive exosomes) contain particles within the established size range for exosomes, 40–150 nm [50] (Fig 1B and 1C). Finally, NanoSight NTA was utilized to quantify exosome isolates (particles/mL). The amount of exosomes released from curcumin-treated PANC-1 cells was not statistically different from the amount of exosomes released from untreated PANC-1 cells (Fig 1D) (p = 0.8371).

### Curcumin detection within exosomes

**Fig 2:**
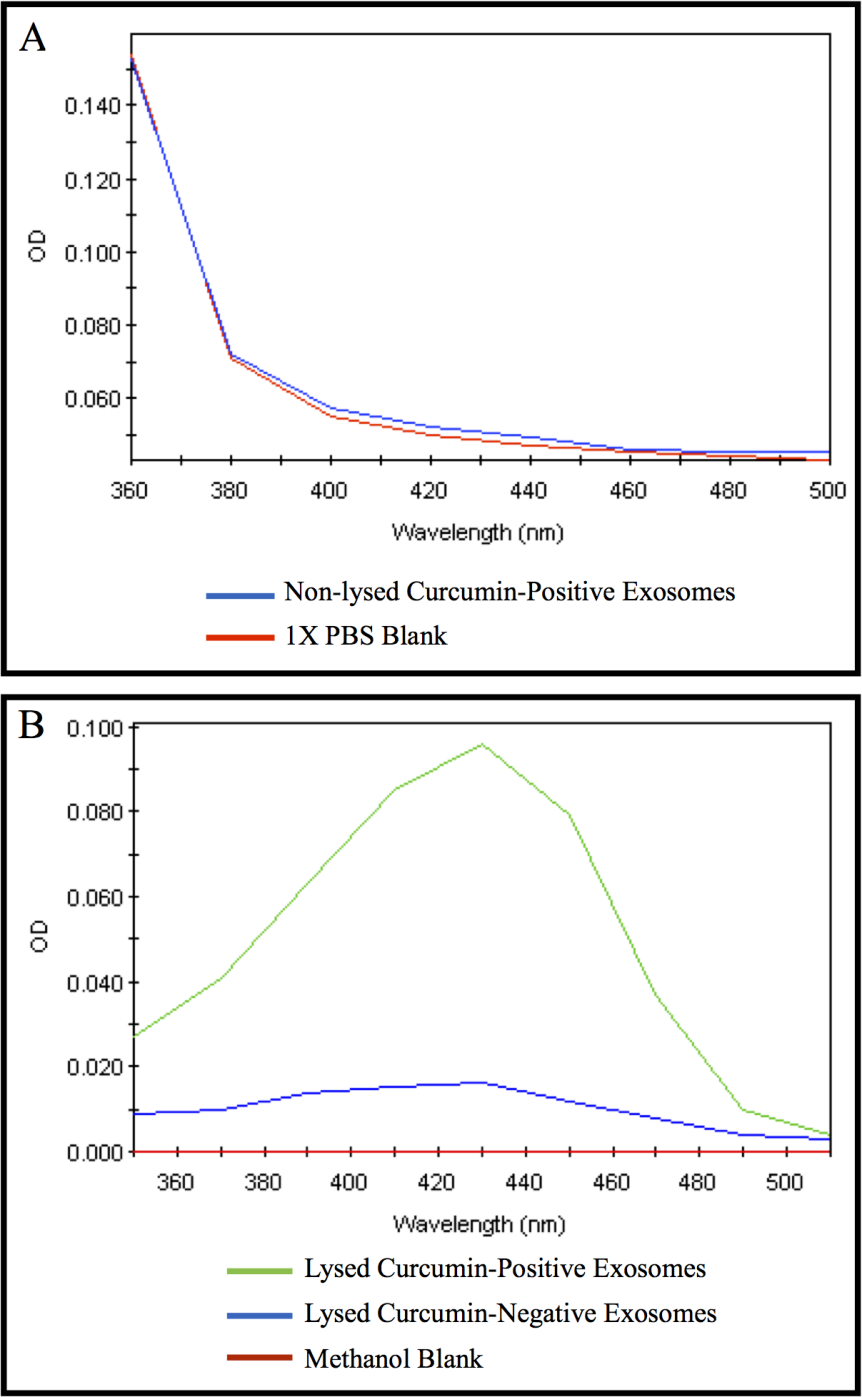
Spectrophotometric detection of curcumin within exosomes from PANC-1 cells. Exosomes were isolated from untreated PANC-1 cells (curcumin-negative exosomes) or PANC-1 cells treated with 50 μM of curcumin for 24 hours (curcumin-positive exosomes). (A) Whole (non-lysed, blue) exosomes from curcumin-treated PANC-1 cells were subjected to spectral analysis compared to vehicle (1X PBS blank, red), in which optical density (OD) at 420 nm was measured. No peak in absorbance was detected at 420 nm from whole (non-lysed) exosomes. (B) Methanol and sonication were used to lyse exosomes from curcumin-treated PANC-1 cells (lysed curcumin-positive exosomes, green) or exosomes from untreated PANC-1 cells (lysed curcumin-negative exosomes, blue). A methanol-only blank (red) was used as a negative control for this assay. A characteristic peak in OD at 420 nm was detected in lysed curcumin-positive exosomes, but not in lysed curcumin-negative exosomes or the methanol-only blank. Data are representative of three independent experiments.

To determine whether curcumin coats the exterior of exosomes, whole exosomes isolated from curcumin-treated PANC-1 cells were subjected to spectral analysis and compared to assay diluent (1X PBS) (Fig 2A). These studies demonstrate a lack of the characteristic absorbance peak corresponding to curcumin at 420 nm. To determine whether curcumin is incorporated into exosomes, exosomes isolated from PANC-1 cells untreated or treated with 50 μM of curcumin for 24 hours were isolated and lysed in 100% methanol. The absorbance peak of exosomes isolated from curcumin-treated PANC-1 cells at 420 nm (lysed curcumin-positive exosomes; green) was compared to exosomes isolated from PANC-1 cells not treated with curcumin (lysed curcumin-negative exosomes; blue) or methanol-only blank samples (red) (Fig 2B). Collectively, these data demonstrate that curcumin is not detectable on the exterior surface of the exosomes but is rather located within the lumen of the exosomes.

### Entry of exosomal curcumin into recipient PANC-1 cells

**Fig 3:**
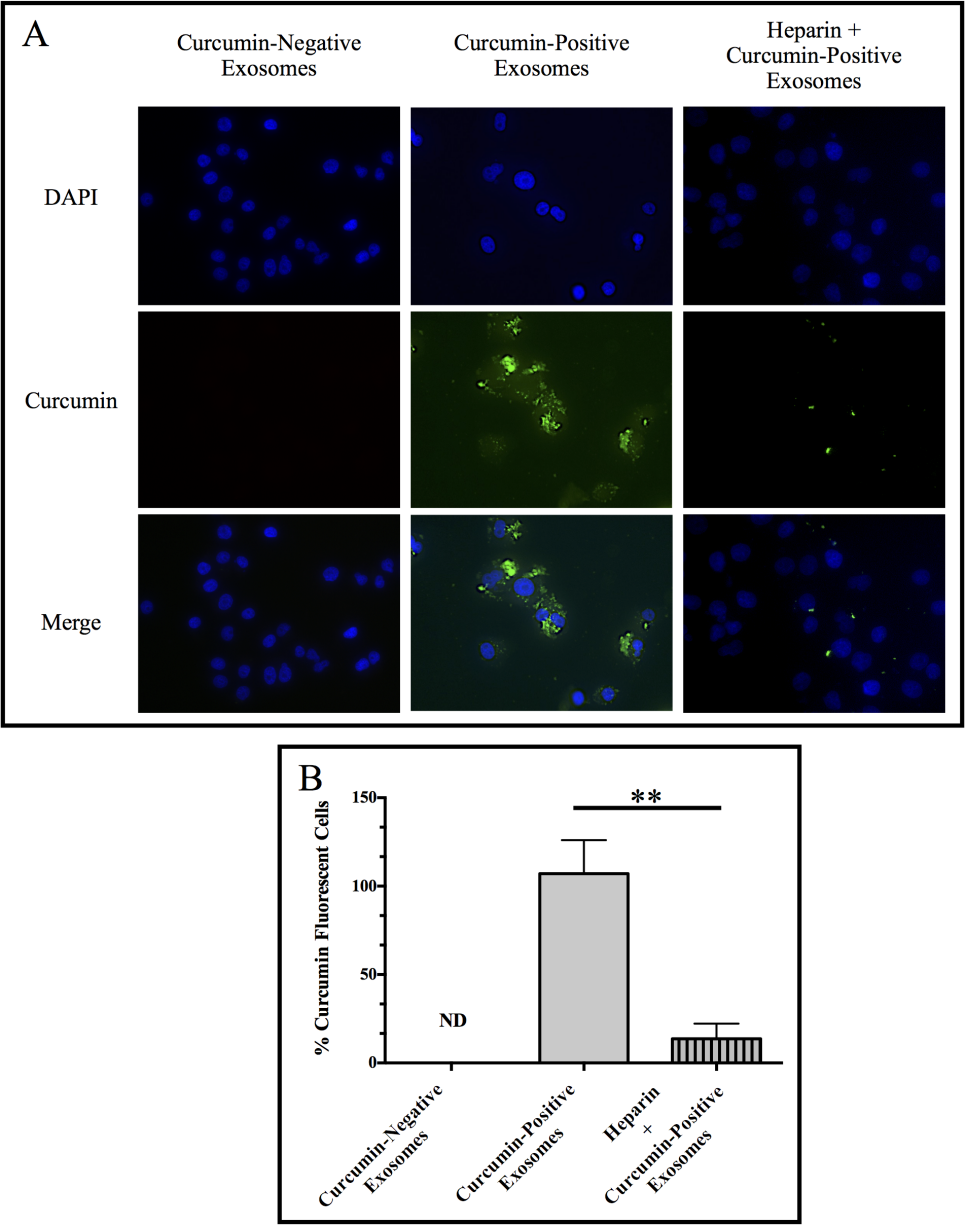
Exosomal curcumin in recipient PANC-1 cells. Naïve PANC-1 cells were co-incubated with exosomes from untreated PANC-1 cells (curcumin-negative exosomes) or exosomes from PANC-1 cells treated with 50 μM of curcumin for 24 hours (curcumin-positive exosomes). In a separate culture, naïve recipient PANC-1 cells were treated with 10 μg/mL heparin to inhibit exosomal binding 30 minutes prior to and during co-incubation with curcumin-positive exosomes (heparin + curcumin-positive exosomes). After 24 hours, cells were washed and stained with DAPI for visualization of nuclei. Curcumin fluorescence (green) and DAPI (blue) were detected by fluorescence microscopy at 40X magnification. (B) Quantification of curcumin fluorescence was performed using the BZ II analyzer software. Data were collected in three separate images per independent experiment, three independent experiments. Data are represented as mean + SEM of three independent experiments, *p<0.05, **p<0.01, heparin + curcumin-positive exosomes versus curcumin-positive exosomes. ND = not detectable.

Our fluorescence microscopy data demonstrate increased curcumin content (green) in PANC-1 cells exposed to curcumin-positive exosomes compared with PANC-1 cells exposed to curcumin-negative exosomes (Fig 3A). The DNA dye DAPI was used to visualize cell nuclei (blue). Furthermore, curcumin content (green) in recipient PANC-1 cells demonstrates a cytoplasmic pattern excluding the nucleus (Fig 3A). Interestingly, if recipient PANC-1 cells were pre-treated with 10 μg/mL heparin, the cytoplasmic detection of curcumin is markedly reduced (p = 0.0054, compared to curcumin-positive exosomes), validating exosomal transfer of curcumin in these studies (Fig 3A and 3B).

### Exosomal curcumin reduces recipient pancreatic adenocarcinoma cell viability

**Fig 4:**
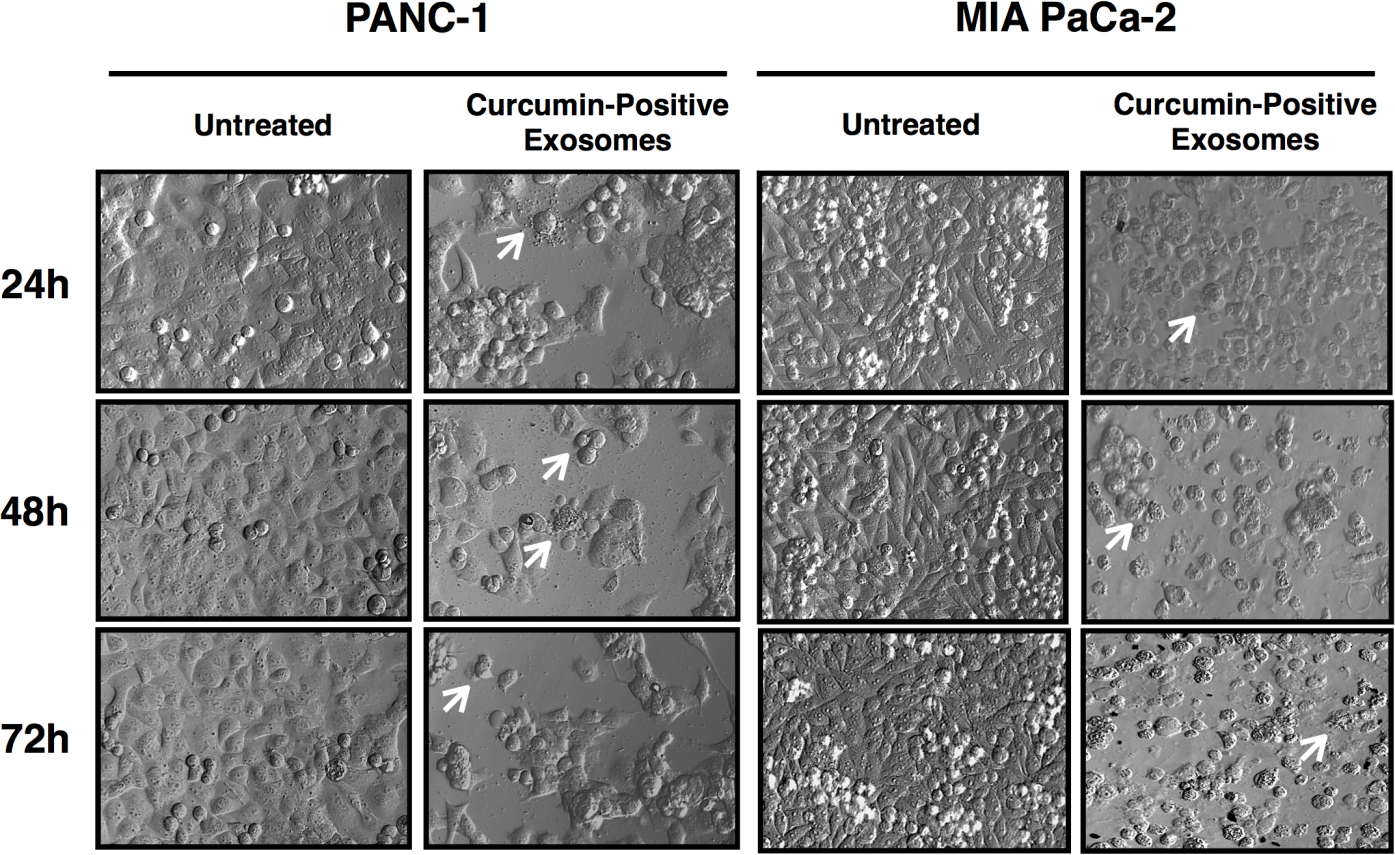
Morphological features of recipient pancreatic adenocarcinoma cells following exposure to exosomal curcumin. PANC-1 and MIA PaCa-2 cells were exposed to curcumin-positive exosomes or exosome-free supplemented DMEM (untreated) for 24, 48 and 72 hours followed by imaging via Hoffman modulation contrast microscopy. White arrows indicate membrane blebs and cell shrinkage, morphological hallmarks of apoptosis. Results depicted represent findings from three independent experiments.

**Fig 5:**
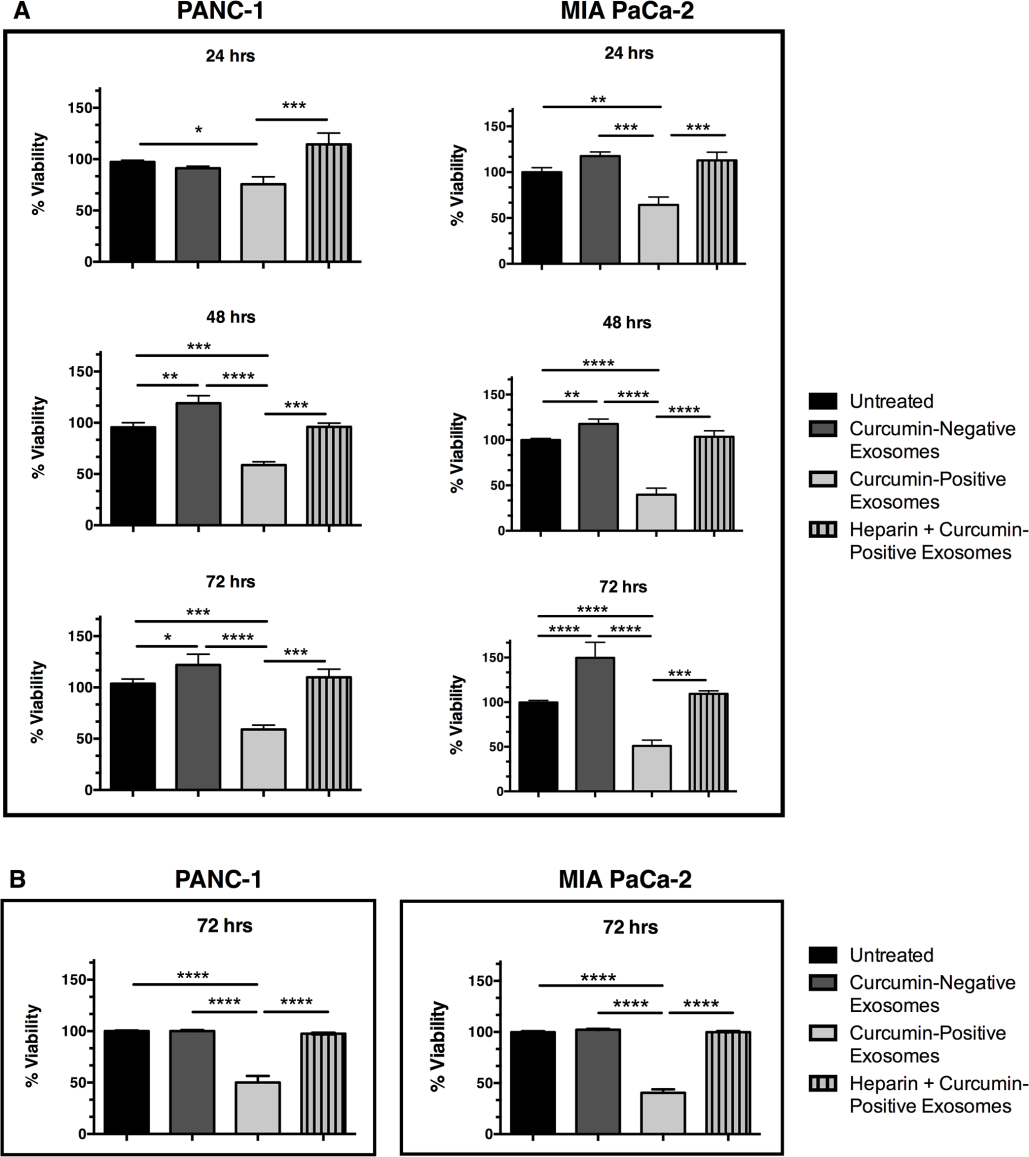
Exosomal curcumin reduces recipient pancreatic adenocarcinoma cell viability. Naïve recipient PANC-1 or MIA PaCa-2 cells were cultured for the indicated times with exosomes isolated from untreated cells (curcumin-negative exosomes) or exosomes isolated from cells treated with 50 μM of curcumin (curcumin-positive exosomes). In a separate experiment, naïve recipient cells were treated with 10 μg/mL heparin prior to and during incubation with curcumin-positive exosomes (heparin + curcumin-positive exosomes). Viability was determined via (A) AlamarBlue and (B) Trypan blue exclusion assays and exosome treatments were compared to naïve cells not exposed to exosomes or heparin (untreated). Data are represented as mean + SEM of three independent experiments, *p<0.05, **p<0.01, ***p<0.001, ****p<0.0001.

The effects of exosomal curcumin on recipient PANC-1 and MIA PaCa-2 cell viability were evaluating using Hoffman modulation contrast microscopy (Fig 4) as well as AlamarBlue and Trypan blue exclusion assays (Fig 5). Cellular morphology following exposure to curcumin-positive exosomes for 24, 48 and 72 hours illustrates key apoptotic morphological hallmarks, including membrane blebbing and cell shrinkage as indicated by white arrows, in both PANC-1 and MIA PaCa-2 cells (Fig 4). Assessment of cell viability using AlamarBlue assays (Fig 5A) demonstrates a significant reduction in viability of pancreatic adenocarcinoma cells exposed to curcumin-positive exosomes compared to untreated controls (p = 0.0116 PANC-1, p = 0.0019 MIA PaCa-2 at 24 hours; p = 0.0002 PANC-1, p = 0.0001 MIA PaCa-2 at 48 hours; p = 0.0003 PANC-1, p = 0.0001 MIA PaCa-2 at 72 hours) as well as compared to exposure to curcumin-negative exosomes (p = 0.0851 PANC-1, p = 0.0004 MIA PaCa-2 at 24 hours; p = 0.0001 PANC-1, p = 0.0001 MIA PaCa-2 at 48 hours; p = 0.0001 PANC-1, p = 0.0001 MIA PaCa-2 at 72 hours). The reduction in cell viability following exposure to curcumin-positive exosomes was abolished by pre-treatment with 10 μg/mL heparin, an inhibitor of exosome binding [47–49] (curcumin-positive exosomes versus heparin + curcumin-positive exosomes; p = 0.0007 PANC-1, p = 0.0008 MIA PaCa-2 at 24 hours; p = 0.0007 PANC-1, p = 0.0001 MIA PaCa-2 at 48 hours; p = 0.0004 PANC-1, p = 0.0001 MIA PaCa-2 at 72 hours). These findings are consistent with results from Trypan blue exclusion assays (Fig 5B), which demonstrate that curcumin-positive exosomes reduce PANC-1 and MIA PaCa-2 cell viability after 72 hours of incubation (p = 0.0001 PANC-1, p = 0.0001 MIA PaCa-2), a phenomenon prevented by pre-treatment with heparin (curcumin-positive exosomes versus heparin + curcumin-positive exosomes; p = 0.0001 PANC-1, p = 0.0001 MIA PaCa-2). Interestingly, an increase in cell viability after exposure to curcumin-negative exosomes was noted in AlamarBlue viability assays (curcumin-negative exosomes compared to untreated control; p = 0.0058 PANC-1, p = 0.0048 MIA PaCa-2 at 48 hours; p = 0.0003 PANC-1, p = 0.0001 MIA PaCa-2 at 72 hours). This is consistent with the notion that tumor-derived exosomes have been shown to deliver cancer-driving factors to recipient cells, promoting aggressive behavior [51, 52]. Thus, our data indicate that curcumin conserves its cytotoxic effects on recipient pancreatic cancer cells after exosomal trafficking.

## Discussion

Patients diagnosed with pancreatic cancer have abysmal survival rates because the current treatment options are not sufficient to completely eradicate the disease. The most effective available therapeutic approach is to resect the pancreas; however, only a minimal percent of patients meet the criteria for surgery due to the lack of effective early detection tools. Additionally, most patients relapse despite intensive post-surgery treatment regimens [53]. The resistance to therapy and tumor aggressiveness observed in pancreatic cancer are related to the effects exerted by the components of the tumor microenvironment [5]. Moreover, these effects are highly dependent on signaling networks driven in part by tumor-derived extracellular vesicles such as exosomes [6–9]. For instance, our group has previously shown that Survivin, a protein highly expressed in cancers and essential for carcinogenesis, is localized in intra-cellular an extracellular pools, and that extracellular Survivin enters cancer cells, increasing proliferation, resistance, and invasive potential [51]. These results are consistent with another study conducted by our laboratory that demonstrate that Survivin is transported out of cancer cells via exosomes [52]. Exosomes have also been shown to transport mutant KRAS proteins to colon cancer cells, increasing tumor growth [54, 55]. These results suggest that exosomes have the ability to modulate the components of the tumor microenvironment via the transfer of bioactive molecules that modulate cancer growth. In addition to transporting cancer-promoting material within the tumor microenvironment, exosomes released from primary tumors have been demonstrated to aid in the formation of a suitable metastatic environment that promotes the transition of non-cancerous cells into pre-cancerous cells [56]. For instance, pancreatic cancer cell-derived exosomes have been shown to prepare pre-metastatic organs for population with cancer cells *in vivo* [57]. These results demonstrate the imperative role of exosomes in metastasis in various cancer types including pancreatic cancer.

Curcumin has been considered a promising therapeutic agent for cancer treatment due to its multi-dimensional anti-cancer properties. For instance, curcumin has been shown to modulate signaling molecules essential for the progression of most cancer types including pancreatic cancer [12, 17, 20]. Additionally, curcumin exhibits synergetic effects with Gemcitabine *in vitro* and *in vivo*. In the context of the clinic, curcumin has a tolerable consumption profile as demonstrated by phase I and II clinical trials [24, 26, 27]. One of the main obstacles to curcumin’s utility in the clinic is low bio-distribution [58]. In response to this, numerous investigations have developed alternative approaches to enhance curcumin delivery [26, 28–36]. These studies demonstrate the potential role of curcumin in pancreatic cancer therapy. However, it is imperative to determine the role of curcumin in the pancreatic cancer microenvironment, particularly in the context of exosomes.

Previous studies have shown that curcumin has a pan-cellular distribution in breast cancer cells [44]. This finding may offer an explanation for curcumin’s multi-dimensional regulatory roles and its capacity to influence various cell signaling pathways. Remarkably, our results indicate that curcumin is able to be packaged into exosomes derived from pancreatic cancer cells treated with curcumin. Furthermore, our work shows that exosomal curcumin enters recipient pancreatic cancer cells, reducing cell viability. Using Hoffman modulation microscopy, we were able to demonstrate that exosomal curcumin induces cell death with morphological features suggestive of apoptosis. This is consistent with previous studies by our laboratory and others on curcumin’s mechanism of action. For instance, curcumin has been shown to downregulate transcription factors linked to cancer progression, including NF-κB and STAT3, resulting in depletion of downstream survival targets including BcL-2, BcL-X_L_, cyclin D1, cIAP1 and Survivin and subsequent apoptotic cell death in pancreatic cancer cells [15, 59]. Our laboratory has recently demonstrated that curcumin also induces apoptotic cell death through reduction of the inhibitors of apoptosis (IAP) Survivin, cIAP1, cIAP2 and XIAP at the protein and mRNA levels in pancreatic cancer cells [60]. Taken together, these findings suggest that exosomal curcumin induces apoptosis in recipient pancreatic adenocarcinoma cells; however, further work must be performed to elucidate the key mediators of exosomal curcumin-induced cell death.

It has been well established that curcumin’s cytotoxic effects on cancer cells are enhanced upon encapsulation of curcumin in synthetic nanoparticles and micelles *in vitro* and *in vivo* [28–33, 35, 36]. Our results demonstrate an approximate 40 to 50% decrease in PANC-1 and MIA PaCa-2 cell viability 72 hours after exosomal curcumin uptake as demonstrated by assays such as AlamarBlue and Trypan blue, which measure cell metabolism and assess cell membrane integrity, respectively. Interestingly, the viability of PANC-1 and MIA PaCa-2 cells was restored after the exposure to heparin, an exosome uptake inhibitor. Consistent with this, our fluorescence microscopy data showed that exosomal curcumin uptake was also impaired by heparin. Together, these findings indicate that the cytotoxicity observed was due to pancreatic cancer cell uptake of exosomes loaded with curcumin.

It is also important to note that PANC-1 and MIA PaCa-2 cell-derived exosomes devoid of curcumin increased viability of recipient PANC-1 and MIA PaCa-2 cells, respectively. This is consistent with the notion that tumor-derived exosomes have a cancer-supportive role in the tumor microenvironment [51, 52]. It is also noteworthy to mention that regardless of exosomal curcumin content, tumor-derived exosomes carry pro-cancerous material [6–9]. However, our results demonstrate that these exosomal components were not an impediment to curcumin’s cytotoxic function after exosomal delivery into recipient pancreatic cancer cells.

In summary, our results provide new evidence of curcumin’s ability to expand its anti-cancer functions from one pancreatic cancer cell to a recipient pancreatic cancer cell with the aid of exosomal transportation. These findings reveal that curcumin’s function may not be restricted to individual tumor cells, but may also be extended to components of the tumor microenvironment such as other tumor cells through exosomes. Exosomes represent a crucial mechanism of communication between the components of the tumor microenvironment and also for preparing future metastatic sites [56]. Thus, our results contribute to a better understanding of the role of curcumin in intercellular communication between pancreatic cancer cells and other components of the tumor microenvironment (vascular smooth muscle, stromal cells or fibroblasts, and immune cells). Collectively, these discoveries highlight the promising role of curcumin as a therapeutic agent for the treatment of pancreatic cancer due to its multi-dimensional anti-cancer properties.
